# Complete Amaurosis

**Published:** 1861-07

**Authors:** 


### The Clinique.

### MARINE HOSPITAL—Service Dr. ISHAM.

Cases reported by A. D. ROUSE, Student of Medicine.

I. *Complete Amaurosis : Recovery.*—Wm. Smith, age 34, native of Sweden, good constitution, temperate habits, was admitted May 19. Has never had any serious illness, but for the last nine years has been troubled, at intervals, with severe attacks of headache, for which he found no relief. During this period of time, his appetite was deranged, and his bowels habitually costive. On the 18th of May, while standing at the helm, he was seized with a severe itching, fulness and burning, with diminution of sight in the right eye; for instance, as he looked at the compass he found his vision variable and defective. By the next day, (that of his admission) these symptoms had subsided, leaving the eye entirely blind. Immediately, the left eye began to be similarly affected, and by the 21st, almost total blindness had occurred, the patient not being sensible of the sudden approach of a lighted candle to the face. Examination revealed the media of the eye healthy, slight congestion of the retina, pupils very sluggish, and unnatural fulness of the eyes; tongue morbidly clean, bowels constipated, appetite poor, pulse 85; diagnosis, amaurosis. This was confirmed by an examination of the patient by the oculist, Dr. Holmes.

Believing that the disease depended upon a congestion of the nervous optic apparatus, produced by disorder of the digestive organs, distinguished by the French under the name of *ébluissement*, the Prof. placed him upon treatment accordingly. Wet cups were repeatedly applied to the temples, and a succession of blisters applied to the forehead, nape of neck, and behind the ears. He was purged with combined doses of calomel, comp. ext. of colocynth, and aloes, repeatedly, and placed subsequently upon the internal use of iodide of potassium in eight gr. doses every 2 hours. His sight was rapidly restored, and June 3d the patient could note the time of day upon the watch.

June 8th—Discharged *cured.*

